# Advanced imaging integration in robotic neurosurgery

**DOI:** 10.1097/MS9.0000000000004484

**Published:** 2025-12-04

**Authors:** Muhammad Zaib, Muhammad Khizar, Fatima Ali, Atiqullah Omerzai, Hasibullah Aminpoor

**Affiliations:** a Faculty of Medicine, Georgian American University, Tbilisi, Georgia; b Faculty of Medicine, Fatima Memorial Hospital College of Medicine and Dentistry, Lahore, Pakistan; c Faculty of Medicine, Kazakh National Medical University, Almaty, Kazakhstan; d Faculty of Medicine, Kabul University of Medical Sciences “Abu Ali Ibn Sina”, Kabul, Afghanistan

**Keywords:** imaging, neurology, neurosurgery, robotics, surgery

## Abstract

Advanced robotic platforms are reshaping neurosurgical practice through the integration of real-time, high-definition imaging. The synergy between intraoperative MRI, 3D tractography, and robotic guidance allows unparalleled precision in procedures such as tumor resection and deep brain stimulation. Augmented reality and artificial intelligence (AI)-assisted visualization now provide surgeons with adaptive, intraoperative feedback, optimizing resection margins while minimizing neural injury. In countries such as the United States, Germany, and Japan, these hybrid systems have demonstrated superior targeting accuracy and reduced operative time, marking a transition toward data-driven, patient-specific neurosurgery. Intraoperative verification of electrode placement and cortical mapping further enhances safety and efficacy. However, disparities in adoption persist globally due to cost, infrastructure, and training limitations. As robotic neurosurgery continues to evolve, transparent AI integration and equitable implementation will be vital. This letter highlights the clinical, technological, and global implications of advanced imaging integration in robotic neurosurgery and aligns with current international standards for transparency and innovation in surgical research.

*Dear Editor,*

Neurosurgical precision remains a core challenge in managing deep-seated or eloquent brain lesions. The integration of advanced imaging with robotic systems represents a transformative evolution, enabling surgeons to navigate complex neural structures with sub-millimetric accuracy. Combined intraoperative MRI, CT, and tractography mitigate human error by maintaining optimal trajectories and minimizing cortical disruption^[1]^. The fusion of these modalities allows real-time spatial verification, enhancing both safety and efficiency in tumor and functional neurosurgery^[2]^.

Recent developments in augmented reality (AR) and artificial intelligence (AI)-assisted imaging have further elevated the capabilities of robotic neurosurgery. By overlaying reconstructed 3D MRI and CT datasets on the surgical field, AR-guided neuronavigation refines operative planning and intraoperative orientation^[3]^. Functional tractography, fused with robotic targeting, preserves critical white matter tracts during resections, reducing postoperative deficits. In hybrid operating suites, intraoperative MRI and CT provide continuous updates that close the feedback loop, allowing immediate assessment of resection margins and electrode placements^[2]^. This real-time adaptability is redefining standards of precision and intraoperative safety.

Clinically, imaging-enhanced robotic systems have demonstrated superior targeting accuracy compared to conventional frame-based stereotaxy^[4]^. Robot-assisted insertion of stereoelectroencephalography electrodes provides broader cortical sampling with higher diagnostic yield^[1]^. Moreover, robotic systems enforce “no-fly zones,” automatically avoiding eloquent cortical and subcortical regions^[1]^. These advances have improved outcomes in deep brain stimulation, epilepsy surgery, and skull base tumor resections^[2]^. The capacity to visualize, adapt, and verify intraoperatively exemplifies the emerging paradigm of personalized neurosurgical intervention.

Globally, adoption reflects both innovation and disparity. Approximately 100 out of 1400 U.S. neurosurgical centers routinely employ robotic systems^[1]^. Germany and Japan lead in precision robotics, with Japan operating over 180 neurosurgical robots^[1]^. China has rapidly expanded robotic stereotactic and functional platforms, while emerging nations such as Nepal established their first robotic neurosurgery programs in 2024^[1]^. Addressing the economic and infrastructural gaps through collaborative training and cost-sharing models will be crucial to ensure equitable access worldwide.

In conclusion, the integration of advanced imaging into robotic neurosurgery represents a pivotal step toward safer, data-driven, and patient-specific care. Future work should emphasize standardized reporting, transparent AI integration, and cost-effectiveness analysis to foster global dissemination. Our work is in line with the TITAN Guidelines on the need for transparency in AI use in health care^[5]^.

## Data Availability

Not applicable.

## Research registration unique identifying number (UIN)

No human subjects were involved, as the type of research being submitted is a letter to the editor.
